# Comparative evaluation of refractive outcomes after implantation of two types of intraocular lenses with different diopter intervals (0.25 diopter versus 0.50 diopter)

**DOI:** 10.1186/s12886-018-0840-0

**Published:** 2018-07-18

**Authors:** Minjung Kim, Youngsub Eom, Jong Suk Song, Hyo Myung Kim

**Affiliations:** 1 Department of Ophthalmology, Korea University College of Medicine, Seoul, South Korea; 2 Department of Ophthalmology, Ansan Hospital, Korea University College of Medicine, 123, Jeokgeum-ro, Danwon-gu, Ansan-si, Gyeonggi-do, 15355 South Korea

**Keywords:** Intraocular lens power calculation, Refractive outcomes, Diopter intervals, Bilateral cataract extraction

## Abstract

**Background:**

Intraocular lenses (IOLs) with different diopter (D) intervals may have different tolerance, and may provide different accuracy of refractive outcome after cataract surgery. The aim of the study is to compare the accuracy of refractive outcome after implantation of IOLs with different D intervals after cataract surgery.

**Methods:**

A total of 80 eyes from 40 patients who underwent phacoemulsification with implantation of a 0.50 D interval Akreos AO IOL in one eye and a 0.25 D interval Softec HD™ IOL in the other eye were enrolled. The percentages of eyes with refractive prediction error within ±0.50 D at one month after surgery were compared. To evaluate the effect of the dioptric errors of the IOL itself on refractive prediction error, the percentage of eyes with refractive prediction error within ±0.25 D of the IOL with a standard deviation (SD) of ±0.40 D was compared with that of the IOL with a SD of ±0.11 D through Monte Carlo simulations.

**Results:**

In this clinical study, the percentage of eyes with refractive prediction error within ±0.50 D by the Haigis formula in the Softec HD™ group (85.0%) was significantly greater than that in the Akreos AO group (57.5%; *P* = 0.027). In Monte Carlo simulations, all percentages of eyes with refractive prediction error within ±0.25 D by the Haigis and SRK/T formulas in the Softec HD™ group were significantly greater than those in the Akreos AO group.

**Conclusions:**

The IOL with a 0.25 D interval was more accurate than the IOL with a 0.50 D interval in predicting refractive outcome after cataract surgery.

**Trial registration:**

Current Controlled Trials KCT0002192, Retrospectively registered (Date of registration: 6 January 2017).

## Background

Intraocular lenses (IOLs) replace the human crystalline lens after cataract extraction by phacoemulsification. The advent of precise optical biometry and IOL power calculation formulas has greatly improved postoperative vision by decreasing refractive prediction error in cataract surgery. [1–3] However, it has always been a challenge for cataract surgeons to enhance refractive outcomes.

There are three main sources of error in IOL power calculation: preoperative estimation of effective lens position, measurement of axial length (AL), and corneal power (determined via keratometry [K]), which contribute to 42, 36, and 22% of errors, respectively. [1, 4] However, several factors such as surgical technique and dioptric power accuracy of the IOL can affect the refractive outcomes. [4] IOL power provided by the manufacturer has an allowed tolerance for power labelling, [5] although the IOL power error is known to contribute less than 1.0% to the total error in postoperative refractive prediction. [4, 6] Most of the IOLs used in cataract surgery are produced at 0.5 diopter (D) intervals. A previous study proposed that tolerance limits of ±0.40 D in the range of 15.5 D to 25.0 D is a suitable international standard for 0.5 D interval IOLs. [5, 7] However, 0.25 D interval IOLs have been developed and used in the IOL industry. Their manufacturer reported that these IOLs within the range of 15.5 D to 25.0 D have an error range of ±0.11 D. [8]

In this study, we hypothesized that implantation of IOLs with 0.25 D intervals, a value that is expected to have strict tolerance limits, would produce more accurate refractive outcomes than would IOLs with 0.50 D intervals after phacoemulsification. To test the hypothesis, this study compared refractive outcomes after implantation of 0.25 D interval IOL in one eye and 0.50 D interval IOL in the other eye in patients with bilateral cataract.

## Methods

### Study population

This prospective study was approved by the institutional review board (IRB) of Korea University Ansan Hospital (IRB number: 2016AS0020) and was registered as a clinical trial at https://cris.nih.go.kr (identification number: KCT0002192). All patients provided signed informed consent to participate in a clinical research study. All research and data collection practices adhered to the tenets of the Declaration of Helsinki and good clinical practices.

**Fig. 1:**
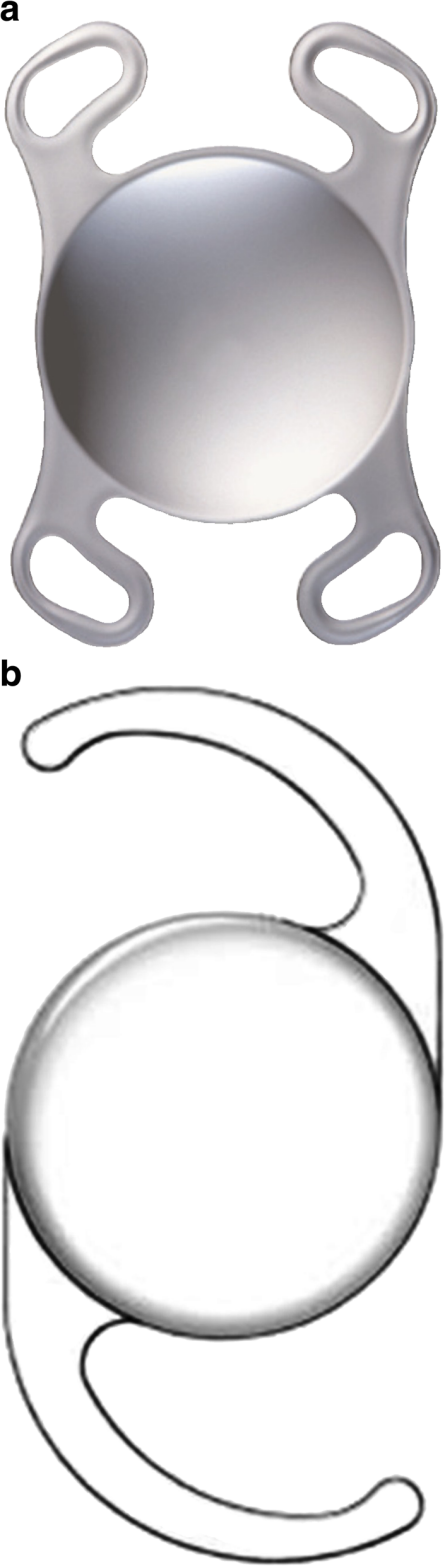
Intraocular lenses used in this study. **a** Akreos AO; **b** Softec HD™

Eighty eyes from 40 patients with bilateral senile cataract who were scheduled to undergo consecutive phacoemulsification and IOL implantation within a period of one to four weeks at Korea University Ansan Hospital between November 1, 2016 and August 31, 2017 were enrolled in this study. The Softec HD™ (Lenstec Inc., St. Petersburg, FL, USA; 40 eyes) IOL with a 0.25 D interval was implanted in one eye of each subject, and the Akreos AO (Bausch & Lomb, Rochester, NY, USA; 40 eyes) IOL with a 0.50 D interval was implanted in the contralateral eye. The RANDBETWEEN(1,2) function in Microsoft Excel (Microsoft Inc., Redmond, WA, USA) was used to randomly decide which IOL would be used in the first eye. The Softec HD™ and Akreos AO IOLs employ similar material and optic design (hydrophilic acrylic bi-aspheric zero aberration with 1.46 refractive index), although their haptic design and overall length are different (Table 1 and Figure 1). We specifically included patients who were scheduled to undergo implantation of IOL with a power range from 15.5 D to 25.0 D because the Softec HD™ IOL provides a 0.25 D interval in this range. Eyes with best corrected visual acuity (BCVA) of 20/40 or better postoperatively were included in this study. Patients with amblyopia, corneal disease such as keratoconus or corneal dystrophy, traumatic cataracts, or a history of previous ocular surgery (e.g., refractive surgery) were excluded. Also excluded from this study were those patients who had undergone complicated ocular surgery (e.g., anterior capsular tears); those who had had any postoperative complications; and those with noticeable postoperative IOL decentration or tilt.

**Table 1:** Characteristics of the IOLs used in this study

Parameter	Akreos AO	Softec HD™
Material	Hydrophilic acrylic (26% water content)	Hydrophilic acrylic (26% water content)
Refractive index	1.46	1.46
Overall length, mm	10.5–11.0	12.0
Optic size, mm	6.00	5.75
Optic design	Bi-aspheric zero aberration	Bi-aspheric zero aberration
Haptic design	Four loop	Modified C
Haptic angulation, degrees	0	0

### Patient examination

Preoperative objective refraction measured with an autorefractor/keratometer (KR-8100; Topcon, Tokyo, Japan) was recorded at the screening visit. Subjective refraction was recorded when autorefraction was not available. Preoperative K, anterior chamber depth (ACD), and AL values were measured with optical biometry using an IOLMaster® 500 (Carl Zeiss Meditec, Jena, Germany). IOL power was calculated using the SRK/T and Haigis formulas of the device. The data-adjusted SRK/T A-constant was calculated using a spreadsheet (Microsoft Excel; Microsoft Inc.), and the data-adjusted a_0_, a_1_, and a_2_ constants for the Haigis formula were calculated with linear regression analysis in order to obtain zero mean numerical error in IOL power prediction. [9–12] The data-adjusted SRK/T A-constant was 118.17 for the Softec HD™ IOL and 118.24 for the Akreos AO IOL. The optimized IOL constants for the Haigis formula (a_0_, a_1_, and a_2_) were 0.565, 0.240, and 0.138, respectively, for the Softec HD™ IOL and 1.706, 0.279, and 0.087, respectively, for the Akreos AO IOL. Postoperative uncorrected distance visual acuity, BCVA, objective refraction measured with an autorefractor/keratometer, and subjective refraction were measured at one month after surgery. The refractionist was masked to type of IOL.

### Surgical technique

All phacoemulsification and IOL implantations were performed by one experienced surgeon (Y.E.) under topical anesthesia with 0.5% proparacaine hydrochloride (Paracaine; Hanmi Pharm, Seoul, Korea). During surgery, a 2.75-mm clear corneal incision was made, and a continuous curvilinear capsulorrhexis slightly smaller in size than the IOL optic was created with a 26-gauge needle and capsulorrhexis forceps. After performing hydrodissection and hydrodelineation, superficial cortex and epinucleus were aspirated with a phaco probe. Then, the nucleus was held by the phaco probe, and the phaco chopper (Nagahara Chopper; ASICO, LLC, Westmont, IL) was placed on the opposite side of the equator of the nucleus. The phaco chopper was pulled and the phaco probe was pushed toward the phaco chopper to divide the nucleus into halves. Then the divided nucleus halves were rotated 90 degrees and divided into quadrants with the same technique. All pieces of segments of the divided nucleus were emulsified and aspirated. An irrigation/aspiration system was used to remove the epinucleus and cortex. The anterior chamber was filled with sodium hyaluronate 1.65% /chondroitin sodium sulfate 4.0% (DisCoVisc; Alcon Laboratories, Inc., Fort Worth, TX), and each IOL was implanted in a capsular bag using an injector system. After removing the ophthalmic viscosurgical device, the wound was sutured with 10–0 nylon, which was removed at postoperative 1 week.

### Preoperative and postoperative medication

Preoperative medication 0.5% levofloxacin hydrate (Cravit®, Santen, Osaka, Japan) every 6 h and 0.1% bromfenac sodium hydrate (Bronuck®, Taejoon Pharm., Seoul, Korea) every 12 h were used for 3 days before surgery. At the day of surgery, 0.5% Moxifloxacin (Vigamox®, Alcon) every 2 h, 0.1% fluorometholone (Flucon®, Alcon) every 6 h, and 0.1% bromfenac sodium hydrate (Bronuck®) every 12 h were used. At the day after surgery, 0.5% Moxifloxacin was used every 6 h, along with the other eye drops used on 1 day for the next 1 month.

### Monte Carlo simulation

To evaluate the effect of the dioptric error of the IOL produced during the manufacturing process on refractive outcome, only IOL power was used as a random variable in Monte Carlo simulation, and it was assumed that no error was caused by the preoperative estimation of effective lens position and/or the measurement of K, ACD, and AL in the IOL power calculation. In order to satisfy this assumption, IOL power, which has zero refractive prediction error, was calculated from preoperative biometry and postoperative spherical equivalent values in each patient. The mean and standard deviation (SD) values were used to generate random variables of IOL power for each patient with a normal distribution using the RAND() and NORMINV functions in Excel (Microsoft Inc.). The mean value of IOL power was defined as the IOL power that has zero refractive prediction error in each patient, and SD was ±0.40 D in the Akreos AO group and ± 0.11 D in the Softec HD™ group. [7, 8] Generated IOL powers for each patient were used to calculate the refractive prediction error during Monte Carlo simulation. This study conducted 20 Monte Carlo simulations: 10 using the Haigis formula and 10 using the SRK/T formula.

### Main outcome measures

The refractive prediction error was defined as the difference between postoperative spherical equivalent and preoperative predicted refraction as determined using two IOL calculation formulas (i.e., refractive prediction error = postoperative spherical equivalent – preoperative predicted refraction). The mean absolute error (MAE) was defined as the mean absolute value of the refractive prediction error, and the median absolute error (MedAE) was defined as the median absolute value of the refractive prediction error. The percentages of eyes that achieved a postoperative refractive prediction error within ±0.25 D, ± 0.50 D, and ± 0.75 D from the preoperative predicted refraction were estimated.

### Statistical analysis

Descriptive statistics for all patient data were obtained using the Statistical Package for Social Sciences version 21.0 (IBM Corp., Armonk, NY, USA). Student’s *t*-tests were used to compare K, ACD, AL, IOL power, and MAE, while Mann–Whitney U tests were used to compare MedAE between the two groups. Chi-square tests were conducted to compare the percentage of eyes that achieved a postoperative refractive prediction error within ±0.25 or ± 0.50 D from the preoperative predicted refraction by the SRK/T and Haigis formulas between the two groups. Results were considered statistically significant if the *P*-value was less than 0.05.

## Results

**Fig. 2:**
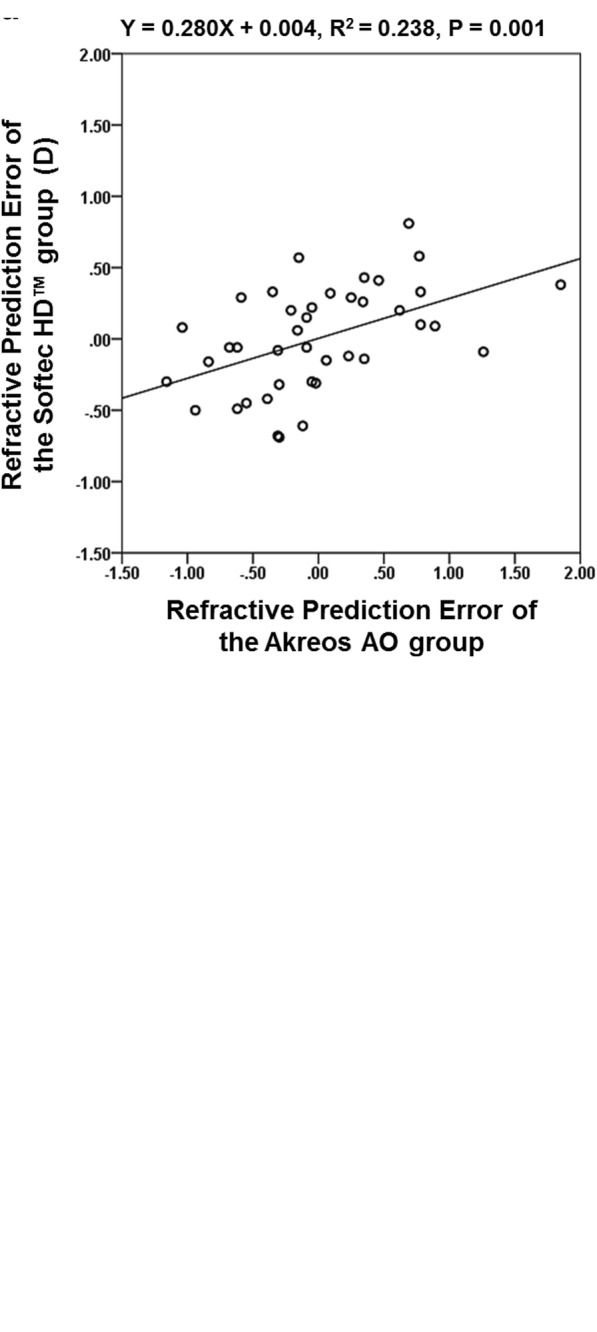
Interocular correlation of refractive prediction error with the Haigis and SRK/T formulas. **a** Haigis formula; **b** SRK/T formula. D = diopters

The mean patient age was 70.2 ± 9.4 years (range: 43–88 years). Fourteen patients (35.0%) were male, and 26 patients were female. The Akreos AO group used 12 different IOLs (power range: 17.0–24.0 D), and the Softec HD™ group used 20 different IOLs (power range: 16.75–24.25 D). There was a significant correlation in the mean K (R^2^ = 0.926, *P* <  0.001), ACD (R^2^ = 0.893, *P* <  0.001), and AL (R^2^ = 0.934, P <  0.001) values between the Akreos AO and Softec HD™ groups. In addition, there was a moderate to strong correlation in refractive prediction error between the two groups (Figure 2). The laterality, mean refractive error, K, ACD, AL, and calculated IOL power values are shown in Table 2.

**Table 2:** Clinical characteristics of the cataract patients and their eyes

Parameter	Akreos AO (n = 40)	Softec HD™ (*n* = 40)	*P*-value^b^
Age, years	70.2 (9.4)		
Sex			
Male, n (%)	14 (35.0)		
Female, n (%)	26 (65.0)		
Laterality			> 0.999^c^
Right eye, n (%)	20 (50.0)	20 (50.0)	
Left eye, n (%)	20 (50.0)	20 (50.0)	
Refractive error, D^a^	− 0.92 (2.34)	−0.88 (2.44)	0.935
Keratometry, D^b^	44.41 (1.37)	44.36 (1.35)	0.880
Anterior chamber depth, mm^b^	3.14 (0.37)	3.17 (0.35)	0.769
Axial length, mm^b^	23.42 (0.70)	23.44 (0.69)	0.907
IOL power, D	20.50 (1.88)	20.37 (1.74)	0.747

Data are mean (SD) except for sex and laterality, which are n (%)

*IOL* Intraocular lens, *D* Diopters, *SD* Standard deviation

^a^Refractive error was measured by an autorefractor/keratometer (KR-8100). Subjective refraction was recorded when autorefraction was not available

^b^Keratometry, anterior chamber depth, and axial length were measured by the IOLMaster® 500

^b^Student’s t-test

^c^Chi-square test

Table 3 shows MedAE and mean refractive prediction error as determined by the Haigis and SRK/T formulas. The MedAE in the Softec HD™ group (0.30 D with the Haigis formula and 0.26 D with the SRK/T formula) was significantly smaller than that in the Akreos AO group (0.35 D with the Haigis formula and 0.37 D with the SRK/T formula; *P* = 0.037 and *P* = 0.049, respectively). The percentage of eyes that achieved postoperative refractive prediction error within ±0.25 D, ± 0.50 D, and ± 0.75 D from the preoperative predicted refraction by the Haigis formula was 32.5, 57.5, and 75.0%, respectively, in the Akreos AO group and 42.5, 85.0, and 97.5%, respectively, in the Softec HD™ group. This percentage within ±0.50 D by the Haigis formula in the Softec HD™ group was significantly greater than that in the Akreos AO group (*P* = 0.027). On the other hand, there was no significant difference in the percentage of eyes that achieved a postoperative refractive prediction error within ±0.50 D from the preoperative predicted refraction by the SRK/T formula between the Akreos AO and Softec HD™ groups (Table 3).

**Table 3:** Comparison of median absolute error and mean refractive prediction error between the Akreos AO and Softec HD™ groups in the Haigis and SRK/T formulas (*n* = 80)

	Akreos AO (*n* = 40)	Softec HD™ (n = 40)	*P*-value
Haigis formula			
MedAE, D^a^	0.35 (0.17: 0.75)	0.30 (0.13: 0.43)	0.037^c^
MAE, D^b^	0.49 (0.40)	0.30 (0.20)	0.009^d^
RE, D (range)	0.00 (−1.16–1.85)	0.00 (− 0.69–0.81)	
± 0.25 D, n (%)	13 (32.5)	17 (42.5)	
± 0.50 D, n (%)	23 (57.5)	34 (85.0)	0.027^e^
± 0.75 D, n (%)	130 (75.0)	39 (97.5)	
> ±1.00 D, n (%)	4 (10.0)	0 (0.0)	
SRK/T formula			
MedAE, D^a^	0.37 (0.21: 0.78)	0.26 (0.12: 0.48)	0.049^c^
MAE, D^b^	0.50 (0.40)	0.33 (0.26)	0.030^d^
RE, D (range)	0.00 (−1.02–1.66)	0.03 (−1.15–1.23)	
±0.25 D, n (%)	13 (32.5)	20 (50.0)	
±0.50 D, n (%)	26 (65.0)	31 (77.5)	0.323^e^
±0.75 D, n (%)	29 (72.5)	37 (92.5)	
> ±1.00 D, n (%)	4 (10.0)	2 (5.0)	

*MedAE* Median absolute error, *D* Diopters, *MAE* Mean absolute error, *RE* Mean refractive prediction error

^a^Values are presented as median (interquartile range)

^b^Values are presented as mean (SD)

^c^Mann–Whitney U test

^d^Student’s t-test

^e^Chi-square test

In the Monte Carlo simulations, the MedAE as determined by the Haigis formula ranged from 0.14 D to 0.27 D, and that determined by the SRK/T formula ranged from 0.14 D to 0.24 D in the Akreos AO group. All MedAEs in the Akreos AO group were significantly greater than those in the Softec HD™ group (range: from 0.04 to 0.06 D in the Haigis formula and from 0.04 to 0.06 D in the SRK/T formula; Tables 4 and 5). Because the MedAE in the Monte Carlo simulations was about half or less than that in clinical study, the percentage of eyes that achieved a postoperative refractive prediction error within ±0.25 D from the preoperative predicted refraction was compared. The percentage of eyes that achieved a postoperative refractive prediction error within ±0.25 D as determined by the Haigis formula ranged from 45.0 to 72.5%, and that determined by the SRK/T formula ranged from 52.5 to 72.5% in the Akreos AO group. This percentage within ±0.25 D in the Akreos AO group was significantly smaller than that in the Softec HD™ group (range: 97.5 to 100.0% in the Haigis formula and 97.5% in the SRK/T formula; Tables 4 and 5).

**Table 4:** Comparison of median absolute error and percentage of eyes that achieved a postoperative refractive prediction error within ±0.25 D by the Haigis formula between the Akreos AO and Softec HD™ groups in Monte Carlo simulation

		Akreos AO (*n* = 40)	Softec HD™ (*n* = 40)	*P*-value
Simulation 1	MedAE, D^a^	0.21 (0.08: 0.27)	0.06 (0.02: 0.09)	< 0.001^b^
	RE < ± 0.25 D, n (%)	28 (70.0)	40 (100.0)	< 0.001^c^
Simulation 2	MedAE, D^a^	0.24 (0.09: 0.35)	0.06 (0.03: 0.10)	< 0.001^b^
	RE < ± 0.25 D, n (%)	22 (55.0)	40 (100.0)	< 0.001^c^
Simulation 3	MedAE, D^a^	0.14 (0.08: 0.29)	0.06 (0.04: 0.09)	< 0.001^b^
	RE < ± 0.25 D, n (%)	29 (72.5)	40 (100.0)	< 0.001^c^
Simulation 4	MedAE, D^a^	0.18 (0.11: 0.33)	0.05 (0.02: 0.07)	< 0.001^b^
	RE < ± 0.25 D, n (%)	26 (65.0)	40 (100.0)	< 0.001^c^
Simulation 5	MedAE, D^a^	0.23 (0.09: 0.33)	0.05 (0.03: 0.10)	< 0.001^b^
	RE < ± 0.25 D, n (%)	22 (55.0)	39 (97.5)	< 0.001^c^
Simulation 6	MedAE, D^a^	0.27 (0.13: 0.37)	0.05 (0.04: 0.08)	< 0.001^b^
	RE < ± 0.25 D, n (%)	18 (45.0)	40 (100.0)	< 0.001^c^
Simulation 7	MedAE, D^a^	0.22 (0.12: 0.39)	0.06 (0.04: 0.11)	< 0.001^b^
	RE < ± 0.25 D, n (%)	23 (57.5)	39 (97.5)	< 0.001^c^
Simulation 8	MedAE, D^a^	0.18 (0.06: 0.31)	0.06 (0.03: 0.10)	< 0.001^b^
	RE < ± 0.25 D, n (%)	24 (60.0)	40 (100.0)	< 0.001^c^
Simulation 9	MedAE, D^a^	0.24 (0.11: 0.34)	0.05 (0.02: 0.10)	< 0.001^b^
	RE < ± 0.25 D, n (%)	23 (57.5)	40 (100.0)	< 0.001^c^
Simulation 10	MedAE, D^a^	0.19 (0.11: 0.35)	0.04 (0.02: 0.09)	< 0.001^b^
	RE < ± 0.25 D, n (%)	23 (57.5)	40 (100.0)	< 0.001^c^

*MedAE* Median absolute error, *D* Diopters, *RE* Mean refractive prediction error

^a^Values are presented as median (interquartile range)

^b^Mann–Whitney U test

^c^Chi-square test

**Table 5:** Comparison of median absolute error and percentage of eyes that achieved a postoperative refractive prediction error within ±0.25 D by the SRK/T formula between the Akreos AO and Softec HD™ groups in Monte Carlo simulation

		Akreos AO (n = 40)	Softec HD™ (n = 40)	*P*-value
Simulation 1	MedAE, D^a^	0.21 (0.11: 0.29)	0.05 (0.04: 0.10)	< 0.001^b^
	RE < ±0.25 D, n (%)	24 (60.0)	39 (97.5)	< 0.001^c^
Simulation 2	MedAE, D^a^	0.15 (0.06: 0.28)	0.06 (0.02: 0.10)	< 0.001^b^
	RE < ±0.25 D, n (%)	29 (72.5)	39 (97.5)	< 0.001^c^
Simulation 3	MedAE, D^a^	0.18 (0.11: 0.32)	0.05 (0.03: 0.07)	< 0.001^b^
	RE < ±0.25 D, n (%)	25 (62.5)	39 (97.5)	< 0.001^c^
Simulation 4	MedAE, D^a^	0.23 (0.13: 0.35)	0.06 (0.03: 0.10)	< 0.001^b^
	RE < ±0.25 D, n (%)	23 (57.5)	39 (97.5)	< 0.001^c^
Simulation 5	MedAE, D^a^	0.16 (0.07: 0.31)	0.05 (0.03: 0.07)	< 0.001^b^
	RE < ±0.25 D, n (%)	25 (62.5)	39 (97.5)	< 0.001^c^
Simulation 6	MedAE, D^a^	0.14 (0.08: 0.29)	0.06 (0.02: 0.09)	< 0.001^b^
	RE < ±0.25 D, n (%)	27 (67.5)	39 (97.5)	< 0.001^c^
Simulation 7	MedAE, D^a^	0.21 (0.11: 0.29)	0.04 (0.01: 0.09)	< 0.001^b^
	RE < ±0.25 D, n (%)	25 (62.5)	39 (97.5)	< 0.001^c^
Simulation 8	MedAE, D^a^	0.19 (0.13: 0.37)	0.06 (0.04: 0.11)	< 0.001^b^
	RE < ±0.25 D, n (%)	26 (65.0)	39 (97.5)	< 0.001^c^
Simulation 9	MedAE, D^a^	0.19 (0.10: 0.33)	0.06 (0.04: 0.09)	< 0.001^b^
	RE < ±0.25 D, n (%)	26 (65.0)	39 (97.5)	< 0.001^c^
Simulation 10	MedAE, D^a^	0.24 (0.07: 0.36)	0.06 (0.05: 0.10)	< 0.001^b^
	RE < ±0.25 D, n (%)	21 (52.5)	39 (97.5)	< 0.001^c^

*MedAE* median absolute error, *D* diopters, *RE* mean refractive prediction error

^a^Values are presented as median (interquartile range)

^b^Mann–Whitney U test

^c^Chi-square test

## Discussion

This study compared the accuracy of refractive outcomes of IOLs with different diopter intervals (0.50 D versus 0.25 D) in cataract surgery and showed that the IOL with a 0.25 D interval had a postoperative spherical equivalent closer to the target refraction predicted by the Haigis and SRK/T formulas than did the IOL with a 0.50 D interval. In addition, more eyes showed refractive prediction error within ±0.50 D with the IOLs with a 0.25 D interval than with the IOLs with a 0.50 D interval. The results of this study suggest that the implantation of IOLs with 0.25 D intervals, which are expected to have strict tolerance limits, would yield more accurate refractive outcomes than implantation of IOLs with 0.50 D intervals after phacoemulsification.

In this study, the percentages of eyes that achieved a refractive prediction error within ±0.50 D were 85.0 and 77.5% as determined by the Haigis and SRK/T formulas, respectively, in the Softec HD™ group. In line with this study, David et al. [13] performed a study involving 291 eyes that underwent cataract surgery with implantation of Softec HD™ IOLs and reported that 72.2% of these eyes achieved a refractive prediction error within ±0.50 D of the target at one month postoperatively. On the other hand, a smaller proportion of eyes with IOLs with a 0.50 D interval (57.5 and 65.0%) achieved a refractive prediction error within ±0.50 D in this study. Similarly, in our previous study that investigated the refractive outcomes of 158 eyes that underwent cataract surgery with implantation of IOLs with 0.50 D intervals, 62.7 and 61.4% of them achieved a refractive prediction error within ±0.50 D according to the Haigis and SRK/T formulas, respectively. [14]

It is common knowledge that several factors affect achievement of accurate refractive outcomes after cataract surgery. These variables include surgical techniques, preoperative measures using biometry, IOL power calculation formulas, optimized IOL constants, and dioptric power accuracy of the implanted IOL. [2–4, 7] In this study, phacoemulsification was performed using the same surgical technique by a single surgeon using the same machine, and the biometry of both eyes was measured by a single examiner under the same environmental conditions. For comparison between the two groups, the Softec HD™ IOL was implanted in one eye of a patient, and the Akreos AO IOL in the contralateral eye. Thus, there should be similar measurement error and effective lens position prediction error in both groups because there is a high degree of interocular symmetry of biometry and refractive prediction error between the two eyes of the same patient. [14, 15] Actually, there was a strong correlation in biometry and a moderate to strong correlation in refractive prediction error between the Akreos AO and Softec HD™ groups in this study. Thus, it seems reasonable to assume that there is only error in IOL power, and that there is no error caused by surgical techniques, preoperative biometry, or IOL power calculation formulas in the Monte Carlo simulations used to evaluate the effect of dioptric power accuracy of the implanted IOL.

In the Monte Carlo simulations of this study, the MedAE of IOLs with an SD of ±0.40 D ranged from 0.14 D to 0.27 D. On the other hand, the MedAE of IOLs with an SD of ±0.11 D ranged from 0.04 D to 0.06 D. According to the these results, if the dioptric power error of the implanted IOL has a normal distribution, the amount of the refractive error at the spectacle plane can be considered to be about half of the SD of the dioptric power error of the implanted IOL. The refractive error of 0.05 D at the spectacle plane may not be clinically meaningful, but an error of 0.20 D may have an effect on IOL power selection. However, it seems that the SD of the dioptric power error of the IOL that was actually produced and used is smaller than the reference value because of the IOL manufacturing technique. Although the dioptric power error of the IOL might be smaller than the international standard tolerance limits of ±0.40 D for 0.5 D interval IOLs, the surgeons performing these operations should know that refractive prediction errors could be caused by the IOL power error itself in the IOL power calculation.

To our knowledge, this is the first prospective, randomized, paired-eye study to consider the different manufacturing tolerances of IOLs to improve refractive outcomes. This study showed that tighter tolerance may contribute to refractions closer to the target value.

There are some limitations to this study. The designs of the haptics of the IOLs used in this study were not the same, although the material, optics design, and optic–haptic angulation were the same. A previous study showed that the nonangulated IOL has less postoperative axial movement than the angulated IOL. [16] The Akreos AO IOL is a single-piece, four-haptic IOL with an overall length of 10.7 mm. In contrast, the Softec HD™ IOL is a single-piece, C loop IOL with an overall length of 12.0 mm. The relatively shorter IOL might not fully support the capsular bag during the early postoperative period and could affect postoperative IOL stability. [17, 18] However, we compared the refractive prediction error at one month postoperatively in order to exclude early postoperative IOL position change caused by capsular contraction. In addition, we additionally conducted Monte Carlo simulations and showed that the greater the dioptric errors of the IOL, the larger the number of refractive prediction errors that occurred.

## Conclusions

In conclusion, the IOL with a 0.25 D interval considered in this study was more accurate than the IOL with a 0.50 D interval in predicting refractive outcome after cataract surgery. Surgeons should keep in mind that refractive errors may also occur due to the dioptric error of the implanted IOL itself when calculating IOL power in cataract surgery.

## Abbreviations

ACD: Anterior chamber depth
AL: Axial length
BCVA: Best corrected visual acuity
D: Diopter
IOLs: Intraocular lenses
IRB: Institutional review board
K: Keratometry
MAE: Mean absolute error
MedAE: Median absolute error
SD: Standard deviation
